# Thinking about the consequences: The detrimental role of future thinking on intrapersonal problem-solving in depression

**DOI:** 10.1371/journal.pone.0289676

**Published:** 2023-08-23

**Authors:** Saima Noreen, Barbara Dritschel

**Affiliations:** 1 Department of Psychology, De Montfort University, Leicester, England; 2 School of Psychology and Neuroscience, University of St Andrews, St Andrews, Scotland; University of Hertfordshire, UNITED KINGDOM

## Abstract

Despite the fact that depressed individuals encounter a multitude of social problems in daily life, research on social problem-solving has largely been dominated by research on interpersonal problems and there is a paucity of research on intrapersonal problems. Intrapersonal problems are linked to one’s subjective psychological functioning and involve managing one’s own feelings and emotions pertaining to the self. Given that depressed individuals exhibit impaired emotion regulation, it is possible that their ability to solve intrapersonal problems may be impaired, especially in relation to future thinking. The aim of this study was to investigate whether future thinking, in the form of thinking about the consequences of a problem being resolved or remaining unresolved has an impact on intrapersonal problem-solving in depression. Forty-five depressed and fifty-four non-depressed participants completed a modified version of the means end problem-solving task (MEPS). In the task, participants were presented with a series of intrapersonal problems and were asked to generate consequences of the problems being resolved or remaining unresolved. Participants were then presented with a positive resolution to each of the problems and were asked to solve the problem to achieve the positive resolution. Following a delay, participants were asked to recall all of the consequences initially generated. Overall, depressed individuals generated fewer-relevant means and less effective solutions to problems than non-depressed participants. Depressed individuals also demonstrated impaired intrapersonal problem-solving following the generation of resolved and unresolved consequences, compared to a baseline condition, where no consequences were generated. These findings suggest that future thinking impairs intrapersonal problem-solving and indicates that a more nuanced approach to future thinking and social problem-solving in depression is needed across different real-life problem-solving contexts.

## Introduction

Impaired social problem-solving is central to the development and maintenance of depression [1–8] and is associated with an increased risk of suicide [9–13]. Studies have also found that enhanced problem-solving abilities are associated with a decreased risk of depression [13, 14]. Social problem-solving, refers to the cognitive behavioural process by which an individual attempts to generate adaptive solutions for problem situations that are encountered in daily life. Social problems, can be interpersonal in nature (i.e., *involve two or more individuals*) or intrapersonal (i.e., *self-focused*). Their resolution involves integrating solutions that alter a situation or an individual’s emotional response to the situation thereby reducing negative consequences and promoting positive consequences [15]. Although research has focused on the role of problem-solving deficits as a risk factor for depression, interventions to improve social problem-solving in depression [16] are lacking. In order to address this gap, it is important to gain a better understanding of the processes underpinning effective problem-solving and how they might be affected by depression.

According to D’Zurilla [17] there are two factors that can influence an individual’s ability to generate effective solutions for social problems, problem orientation and problem-solving skill. Problem-orientation refers to one’s own awareness of the problem, their assessment of their own ability to solve the problem and their beliefs about the effectiveness of their proposed solutions in solving the problem. Research has found that the way in which an individual deals with a problem has a subsequent impact on their well-being [18]. For example, it has been found that individuals with a negative problem orientation (i.e., hold unfavourable views about their problem-solving skills which act as a barrier against active problem solution) were more likely to feel depressed and hopeless after encountering social problems [19]. It has also been found that problem orientation is a predictor of depression, with depressed individuals holding a more negative problem orientation than non-depressed individuals [13, 20, 21]. Problem-solving skill refers to an individual’s ability to rationally identify problems, define them appropriately, generate and execute solutions for the problem and then monitor the effectiveness of the solutions [22]. There is considerable evidence for social problem-solving deficits being related to depression [1, 2, 23–25]. Indeed, research has found that depressed individuals generate fewer steps to solve a problem and their solutions are less effective than non-depressed controls [2, 26].

Another factor that is associated with impaired social problem-solving in depression is rumination [27], and its effects on problem-solving have been found to be independent of depression [29]. Depressive rumination is broadly defined as a passive self-focused and recurrent form of negative thinking that centres on one’s symptoms, feelings, causes and consequences of depressed mood [27, 28] and has been implicated in both the onset and maintenance of depression ([29]; see [30] for a review on rumination). Rumination impairs social problem-solving [31] by making depressed individuals more pessimistic. Engaging in repetitive abstract negative thinking, the hallmark of rumination, also makes specific relevant information less accessible, making it more difficult for depressed individuals to solve problems [25, 32, 33].

In more recent years, the importance of future thinking for problem-solving has been highlighted. Future thinking, more broadly, is the ability to represent what might happen in the future. According to Szpunzar, Spreng & Schacter [34] there are four modes of future thinking, which are: simulation, prediction, planning and intention. These four modes of future thinking map on to both semantic and episodic knowledge. Future episodic simulation is the ability to envision possible future events and has a strong adaptive value, allowing one to consider potential consequences prior to acting, and being able to override current short-term needs in favour of long-term goals [35, 36]. Future-directed thoughts are ubiquitous in daily life [37] and play an important role in decision making, emotion regulation and problem-solving [38–43].

Future episodic simulation in social problem-solving has an important adaptive function because it involves combining different types of information in memory and simulating novel conditions or situations [44, 45], which may in turn, aid problem-solving by allowing individuals to generate novel solutions. This facilitation is consistent with research which has found that the ability to retrieve specific rather than general or categoric memories is associated with enhanced problem-solving [23, 46]. Specific memories contain more detailed information which is useful for generating problem-solving solutions [23, 47]. Goddard et al [23] for example, demonstrated that there was a positive association between the ability to retrieve specific memories on the autobiographical memory cueing task (AMT) and the production of more effective solutions on the means end problem-solving task (MEPS; [8]) for non-depressed participants. This relationship was absent in the depressed group, however, who instead spontaneously retrieved more overgeneral categoric memories during problem-solving, which was related to generating less effective solutions.

Research on future thinking in depression has found that depressed individuals anticipate a greater number of future threat-related events, expect negative consequences to occur more frequently and generate and anticipate fewer positive events [48–50]. It has also been found that a reduced ability to imagine positive future events is associated with hopelessness in depression and parasuicide [51].

More recently, the impact of future episodic simulation on social problem-solving in depressed individuals has been investigated, which has shown that thinking about the potential consequences of a social problem being resolved or remaining unresolved has an impact on depressed individuals’ ability to subsequently solve the problem [2]. For example, Noreen and Dritschel [1] presented depressed and non-depressed participants with six scenarios of hypothetical social problems. In the first stage of the study (i.e., consequence generation stage), participants were asked to generate four consequences of the problem being resolved (i.e., *ending with a positive resolution for all involved*) for three of the scenarios and to generate four consequences of the problem remaining unresolved (i.e., *ending without a positive resolution being reached*) for the remaining three scenarios. In the problem-solving phase, participants were then presented with some of the problem-solving scenarios (i.e., two scenarios they previously generated resolved consequences for, two they generated unresolved consequences for, and two new scenarios where no consequences were generated, thus representing a baseline measure of problem-solving) and the resolutions to these problems. They were then asked to solve the problems in order to achieve the positive resolutions described. Finally, in the consequence recall phase, participants were presented with the six problem scenarios presented initially (i.e., in the consequence generation phase) and were asked to recall all of the consequences that they had previously generated.

The study found that depressed participants, compared to non-depressed participants were less successful at solving those problems that they had previously generated unresolved consequences for, as well as, problems in a baseline condition where no consequences were generated. Furthermore, depressed participants were also less successful at solving those problems that they had previously generated unresolved consequences for, compared to problems in the baseline condition. Depressed participants also remembered significantly more of the unresolved than the resolved consequences that they had previously generated. Interestingly, it was found that depressed participants were as successful as non-depressed participants when solving those problems, they had previously generated resolved consequences for. These findings are clinically important and suggest that encouraging depressed individuals to think about the consequences of a problem being resolved may subsequently improve their ability to solve the problems.

Whilst these findings have provided important insights into the factors that influence social problem-solving, it is unclear whether the relationship between depression, social problem-solving and future episodic simulation applies to the vast array of social problems that depressed individuals experience in everyday life. Traditionally, research has treated social problem-solving as a single dimension, with the majority of studies focusing on interpersonal problem-solving [1, 23] even though intrapersonal problems are frequently encountered in daily life. Moreover, the few studies that have looked at both interpersonal and intrapersonal problem-solving have done so by grouping the two types of social problems together [52], thus potentially obscuring important qualitative differences between inter and intrapersonal problems.

As interpersonal problem-solving focuses on generating adaptive solutions to problematic situations in everyday life that involves others, these problems are linked to one’s social functioning and the ability to build and maintain positive relationships. Intrapersonal problems, however, focus on internal problems faced by the individual and are thus linked to one’s subjective psychological functioning and the ability to manage one’s own feelings and emotions pertaining to the individual self [53]. Whilst both forms of problem-solving may be related, with the way that an individual views themselves possibly influencing how they approach social interactions and vice versa [54] they may also be distinct.

Intrapersonal problem-solving for depressed individuals may be more challenging than interpersonal problem-solving given that one of the main features of depression is the effect on how people think about themselves. Indeed, one symptom of depression is the increase in self-focused attention [55]. It has been found that people with depression spend more time thinking about themselves and find it difficult to turn their attention to events unrelated to themselves [56–58]. This internalised focusing not only captures attention but also leads to a negative conception of the self. This is consistent with Beck’s [59] cognitive theory which suggests that a negative view of the self, the world and the future are central features of depression. These views lead to maladaptive interpretations of stressful life events and negative inferences being made. Specifically, it has been found that depressed individuals will generate internal attributions to negative events and to stable and global causes leading to detrimental consequences, such as, feeling hopeless about themselves, perceiving themselves to be incompetent and lacking control [60, 61]. However, for interpersonal problems it is more likely that both internal and external attributions will be made as interpersonal problem-solving involves other individuals. Thus, there may be less self-focus and internalised thinking for these problems. Given that internalised dysfunctional thinking about the self is an important feature of depression, it is possible that these cognitive distortions may act as a barrier to prevent the generation of selective and novel solutions to intrapersonal problems.

Greater self-focus and internalisation may also result in self-discrepant thinking having more profound consequences for intrapersonal versus interpersonal problems. Research has found that self-discrepant thinking, which is the difference between thinking about one’s ideal versus one’s actual self, has an impact on mood [62]. Specifically, it has been found that when depressed individuals recall positive autobiographical memories that are discrepant with the current self, their mood deteriorates. As past and future thinking are related [63] it may be possible that thinking about intrapersonal problems being resolved in a positive manner invokes more self-discrepant thinking than interpersonal problems as they focus more heavily on the self. Self- discrepant thinking in depression results in lower mood which in turn is associated with a negative problem orientation [64].

Furthermore, it is also possible that depressed individuals, compared to non-depressed participants may experience more intrapersonal problems in their everyday life which they are unable to solve effectively. Difficulties in solving these problems may contribute to the maintenance of their depressed mood state and learned helplessness about the ability to solve problems. Thus, identifying whether depressed individuals show deficits in intrapersonal problem-solving and how these deficits interact with future episodic stimulation represents an important next step in furthering our understanding of the interplay between problem-solving, future episodic simulation and depression. Having a more nuanced understanding of whether positive future thinking can help intrapersonal problem-solving in depression creates the potential to develop more effective interventions.

The aim of this study was to investigate whether future episodic stimulation, in terms of thinking about the potential consequences of a problem being resolved or unresolved aids intrapersonal problem-solving in depression. To this end, we implemented the study procedure employed in our previous research [1] examining the impact of future episodic stimulation on solving interpersonal problems in depression but used novel intrapersonal problems. In the current study depressed and non-depressed participants were presented with a series of hypothetical intrapersonal problems. In the consequence generation phase, participants were presented with six problems. For three of the problems, they were asked to generate four consequences for each problem being resolved, and for the remaining three problems participants were asked to generate four consequences for each of the problems remaining unresolved. Participants then underwent the problem-solving phase, whereby they were presented with some of the problem scenarios (i.e., two scenarios they previously generated resolved consequences for, two they generated unresolved consequences for, and, two new scenarios where no consequences were generated, thus representing a baseline measure of problem-solving) and a positive resolution to the problem and were asked to generate the steps they would take to solve the problem to achieve the positive resolution. Following a distractor task, the recall generation phase took place. In this phase, participants were presented with the six problem scenarios presented in the consequence generation phase and were asked to recall all the consequences that they had previously generated.

We predicted that for the baseline condition, the depressed participants would generate less effective solutions and report less relevant-means (i.e. steps) for solving the intrapersonal problems compared to the non-depressed controls. We also predicted that for the unresolved condition, the depressed participants would generate less effective solutions and report less relevant-means for solving the intrapersonal problems compared to the non-depressed participants. For the resolved condition, based on previous findings [1. 2] we predicted that the depressed and controls would show no differences in generating solutions. As rumination impacts problem-solving, we also examined whether rumination has an impact on the relationship between future episodic stimulation and intrapersonal problem -solving in depression, independent of mood. It is possible that rumination may increase internalised self-focused thinking which may impair intrapersonal problem-solving.

## Method

### Participants

In order to allocate participants in the depressed and non-depressed control groups, one hundred and thirteen participants (51M & 62F; age M = 23.41; SD = 3.46) took part in the initial assessment. Participants were recruited from Goldsmiths, University of London and were paid £5 per session for their participation. Participants completed the Mini-International Neuropsychiatric Interview-Plus (MINI-Plus; [65]) and the Beck Depression Inventory-II (BDI-II; [66]). The MINI-Plus was administered by a trained researcher with diagnosis status verified by a second trained researcher. Participants were classified as depressed if they met the criteria for current major depressive disorder on the MINI-Plus and scored at least 15 on BDI-II, and non-depressed controls, if they reported having no current or past Axis One disorders (such as, anxiety disorders, psychotic disorders, mood disorders, substance use disorder etc.) and scored 5 or below on the BDI-II. These criteria resulted in 99 participants taking part in the main session of the study, which took place 7–14 days after the initial screening. Forty-five depressed participants (19M & 26F; age M = 20.44; SD = 3.46) and fifty-four non-depressed controls (21M & 33F; age M = 20.93; SD = 3.32) took part in the main study. The current sample size slightly exceeded the required sample size (N = 66), determined by a priori power analyses conducted using GPower3 [67], (power 1 - β err = .95, d = .20 and α = .05), but was similar to the sample size used in recent relevant research (N = 86 [1]). Thirty-nine participants from the depressed group were educated to degree level (e.g., Bachelors, Masters or PhD), 5 were educated to college level (e.g., A-Level or equivalent) and 1 was educated at school level (e.g., GCSE or equivalent). Forty-four participants from the non-depressed group were educated to degree level, 7 were educated to college level and 3 was educated at school level. The depressed group consisted of 11 participants that met criteria for panic disorder, 9 for social phobia, 2 for bulimia, 12 for dysthymic disorder and 9 for mixed depression and anxiety. Furthermore, 19 also reported taking antidepressant medications in the past and 14 had a history of past depression. Our participants were comprised of a range of ethnic backgrounds with 41 White British, 27 Black British, African or Caribbean and 31 from Minoritised British Pakistani, Bengali and Indian groups. Ethical approval was obtained by Psychology Ethics Committee, Goldsmith, University of London, with written consent obtained for all participants prior to them taking part in the study.

### Materials

In order to allocate participants into depressed and non-depressed groups, a trained researcher conducted a structured psychiatric interview using the Mini-International Neuropsychiatric Interview-Plus 5.0.0 (MINI-plus) to assess depression. The MINI-Plus is a structured diagnostic interview that is used to determine the most common psychiatric disorders according to axis 1 DSM-V and the International Classification of Diseases and Related Health Problems (ICD-10) [65, 68]. It thus screens for mood disorders (e.g., depression, dysthymia), anxiety disorders (e.g., panic disorders, social phobia, specific phobia, generalised anxiety disorder, posttraumatic stress disorder) and substance use (e.g., alcohol dependence). The MINI-plus takes between 15–30 minutes to administer and is specifically designed to be used in clinical practice and research. MINI has been found to demonstrate excellent reliability [65, 68, 69].

The Beck Depression Inventory-II (BDI-II; [66]) was used to assess the severity of the affective (e.g., feelings of sadness, anhedonia), somatic (e.g., appetite disturbance, sleep problems) and cognitive symptoms (e.g., suicidal thoughts, pessimism) of depression and to allocate participants into depressed or non-depressed groups. BDI-II is a self-report measure that consists of 21 items, with each item having a 4-point grade scale scored from 0–3. Total scores range from 0–63, with higher scores indicating more severe depression symptoms. The BDI-II is widely used and has been extensively researched as a self-report measure for assessing depressive symptomology in both psychiatric and non-psychiatric (or healthy) populations [66, 70]. In this study we found BDI-II to be very reliable (21 items; α = .97).

The State-Trait Anxiety Inventory (STAI; [71]) was used to assess both the state (situational) and trait (dispositional) components of anxiety. STAI is comprised of two 20 item self-report questionnaires that either record the presence or absence of anxiety symptoms on a 4-point likert scale, scored from 1 to 4. Total scores range from 20–80 on each questionnaire with higher scores indicating more severe anxiety symptoms. It is widely reported that STAI has excellent psychometric properties with good reliability and validity [72] and is an appropriate measure for studying anxiety in both clinical and research settings [73]. In this study we found STAI to be very reliable (state 20 items; α = .94; trait 20 items; α = .96).

The Ruminative Response Scale (RRS: [74, 75] was used to assess how individuals respond to feelings of sad mood. The RRS is comprised of 22 items that are focused on the self (e.g., thinking about your shortcomings), on symptoms (e.g., difficulty in concentrating) or the consequences of depressed mood (e.g., analysing events to understand why you are depressed). Participants indicate how often they engage in responses using 4- point likert scales with scores from 1 to 4. Total scores range from 22–88, with higher scores indicating greater levels of rumination. The RRS has been found to have good test-retest reliability and validity [74–76]. In this study we found RRS to be highly reliable (22 items; α = .94).

In order to investigate intrapersonal problem-solving, we developed eight hypothetical scenarios. Each scenario featured a hypothetical problem that may be encountered by an individual in their daily life (such as, *not saving your work on a computer*, *needing to lose weight*, *feeling worried about a presentation*, *feeling insecure about your appearance*) and a positive resolution to the problem. The scenarios were taken from a larger collection of 14 hypothetical scenarios that we developed and piloted. In the pilot study, 48 participants (20M, 28F, Mean age = 22.27, SD = 4.59) were presented with the 14 scenarios and were asked to rate each scenario for openness (with 1 = not open at all (i.e. completing a problem using a fixed sequence of steps that must be performed in a given order to solve the problem), and, 7 = very open (i.e. there are many possible solutions which can be used to solve the problem)) and difficulty in solving the problem (with 1 = very easy, and, 7 = very difficult). Half the participants were then asked to generate resolved consequences for the first block of 7 scenarios and unresolved consequences for the second block of 7 scenarios, whilst the other half of the participants generated unresolved consequences for the first block of scenarios and resolved consequences for block 1 of the scenarios. From the results of the pilot study, we selected eight scenarios that were matched on openness, difficulty and the number of resolved and unresolved consequences generated (additional information on the pilot study can be obtained from the authors upon request). (See scenarios in the supplementary materials section).

### Procedure

**Fig 1:**
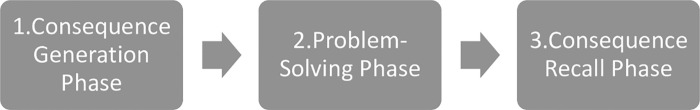
A diagram of the modified MEPS procedure.

In the first session, participants initially completed the MINI-Plus interview, BDI-II, STAI and RRS measures. In the second session, in line with Noreen and Dritschel (2022), participants completed a modified version of the MEPS which consisted of three main phases; consequence generation, problem-solving and consequence recall phase. See Fig 1.

#### Consequence generation phase

Participants were initially presented with six of the eight hypothetical problems and were asked to generate consequences (i.e., the short- and long-term outcomes) of the problem being resolved or remaining unresolved. Specifically, each problem was presented for 4 minutes and participants were asked to generate four consequences for each of the problems being resolved or remaining unresolved. For three of the problem scenarios, participants were asked to generate consequences of the problem being resolved and for the remaining three problem scenarios participants were asked to generate consequences of the problem remaining unresolved. The presentation of the scenarios was fully counterbalanced, with no two resolved or unresolved scenarios appearing consecutively.

#### Problem-solving phase

Participants were then presented with the scenarios and the positive resolutions and were asked to describe the steps that they would take to solve the problem and achieve the positive resolution described.

Participants were presented with each scenario and positive resolution and given 4 minutes to generate the steps to reach the positive ending described. Participants were presented with six of the eight scenarios, which consisted of two scenarios that they had previously generated resolved consequences for and two scenarios that they had previously generated unresolved consequences for. The other two scenarios were not presented in the consequence generation phase and instead represented a baseline measure of problem-solving. Participants were instructed to focus on solving the problem in order to achieve the positive resolution described, irrespective of whether they had previously generated resolved or unresolved consequences.

#### Consequence recall phase

Following a 10-minute distractor task that involved participants completing a series of mathematical problems, participants were then presented with the same six scenarios from the consequence generation phase and were asked to recall all of the consequences that they had generated previously. Specifically, participants were instructed to recall all of the consequences as accurately as possible. Participants were presented with each scenario and were given 4 minutes to recall all four consequences. The scenarios consisted of four scenarios that they had previously attempted to solve (two scenarios that involved generating resolved consequences for and two scenarios that involved generating unresolved consequences for). The other two scenarios were not presented in the problem-solving phase and instead represented a baseline measure of consequence recall (i.e., uninfluenced by problem-solving). Participants were first asked to generate the consequences for the baseline scenarios, followed by the unresolved then the resolved scenarios. The scenarios were all counterbalanced for the consequence generation and problem-solving phases across participants.

1. In this phase six hypothetical problems are presented.
2. In the problem-solving phase six hypothetical problems are presented (for two of the problems participants previously generated consequences of the problem being resolved, for the other two problems participants previously generated consequences of the problem being unresolved and the final two problems were not presented in the consequence generated phase, thus representing a baseline measure of problem-solving.
3. In this phase six hypothetical problems are presented (identical to those presented in the consequence generation phase) and participants are asked to recall all the consequences previously generated.

### Scoring

We scored the MEPS on two main dimensions: the number of relevant means (i.e., the number of active problem-solving steps proposed) and the effectiveness of the solutions proposed (with 1- not at all effective and, 7 = extremely effective). Effective solutions maximised positive consequences and minimised negative consequences. The ratings were scored by an independent coder (blind to the group status of the participants), as well as a second independent rater. The second rater was trained by the four-step process developed by [51] and scored 40% of the solutions in order to validate the scoring. Inter-rater reliability was calculated using Intra-class correlation coefficient using a two-way mixed effects model. There was a high degree of agreement between raters for both relevant means (average ICC = .97, *F*(236, 236) = 33.70, *p <* 0.001), and effectiveness (average ICC = .95, *F*(236, 236) = 21.95, *p <* 0.001).The split half reliability for MEPs was also found to be good (α = .70).

Recall accuracy for the consequences was calculated by comparing the consequences generated in the consequence generation phase with the consequence recall phase. Each of the consequences were scored as correct if the descriptions corresponded to the descriptions initially generated and could be identified as referring to the same point/consequence. Only minor variations in wording were accepted as being correct (e.g., ‘*getting a bad mark for the presentation’* and ‘*getting a poor mark’* would be scored as correct)

## Results

### Participant characteristics

Analysis of participants characteristics demonstrate that the depressed and non-depressed groups did not differ significantly with respect to age. The depressed group did, however score significantly higher on depressive symptoms, rumination, and state and trait anxiety, compared to non-depressed participants. See Table 1.

**Table 1:** Mean performance indices and significant differences on participant characteristics and mood measures, as a function of group (standard deviations are presented in parentheses).

	Depressed Group Mean (SD) (N = 45)	Non-Depressed Group Mean (SD) (N = 54)	Significant differences across Depressed & Non-Depressed groups
**Age** *	20.44 (3.46)	20.93 (3.32)	*t*(97) = .70, *p =* .24, *d =* .14
**BDI-II**	29.40 (9.12)	2.46 (1.30)	*t*(45.49) = 19.64, *p <* .001, *d =* 4.14
**RRS**	62.51 (7.93)	38.35 (12.71)	*t*(90.36) = 11.53, *p <* .001, *d =* 2.28
**STAIT**	49.78 (8.62)	34.52 (10.66)	*t*(97) = 7.73, *p <* .001, *d =* 1.57
**TRAIT**	53.40 (10.80)	34.0 (10.41)	*t*(97) = 9.07, *p <* .001, *d =* 1.83

* In years; BDI-II = Beck Depression Inventory II; RRS = Rumination Response Scale; STAIT = Spielberger State Inventory; TRAIT = Spielberger Trait Inventory.

### Intrapersonal problem-solving: Relevant means

In order to investigate differences in intrapersonal problem-solving ability, we conducted a 2 (group: depressed vs. non-depressed) x 3 (condition: resolved vs. unresolved vs. baseline) mixed design ANOVA to explore the number of relevant means (i.e., steps taken) to reach the proposed solution. Our analysis found a main effect of group; *F* (1, 97) = 143.43, *p <* .001, *η*^*2*^ *p* = .60, and, condition, *F* (1, 97) = 17.99, *p <* .001, *η*^*2*^ *p* = .16. We also found a significant group by condition interaction, *F* (2, 97) = 7.90, *p <* .001, *η*^*2*^ *p* = .08, with subsequent Bonferroni adjusted (*p =* .008) post hoc tests revealing that non-depressed participants proposed significantly more steps compared to depressed participants to reach the proposed solution for the baseline, *t*(97) = 4.53, *p <* .001, *d =* .92, resolved, *t*(97) = 8.46, *p <* .001, *d =* 1.70, and unresolved conditions, *t*(97) = 10.89, *p <* .001, *d =* 2.19. Subsequent pairwise analysis revealed that depressed participants proposed significantly more steps in the baseline than the resolved, *t*(44) = 4.77, *p <* .001, *d =* .88, and unresolved conditions, *t*(44) = 6.61, *p <* .001, *d =* 1.27. There were no differences, however, between the number of steps proposed in the resolved and unresolved conditions, *t*(44) = 1.63, *p =* .06, *d =* .30. For the non-depressed participants we found that there were no differences between the number of steps proposed in the baseline, resolved, *t*(53) = 1.22, *p =* .11, *d =* .24, and, unresolved conditions, *t*(53) = 1.41, *p =* .08, *d =* .25. Furthermore, there were no differences, between the number of steps proposed in the resolved and unresolved conditions, *t*(53) = .05, *p =* .48, *d =* .01.

### Intrapersonal problem-solving: Effectiveness

In order to investigate differences in intrapersonal problem-solving ability, we conducted a 2 (group: depressed vs. non-depressed) x 3 (condition: resolved vs. unresolved vs. baseline) mixed design ANOVA to explore the effectiveness of proposed solutions. We found a significant main effect of group; *F* (1, 97) = 200.25, *p <* .001, *η*^*2*^ *p* = .67, and, condition, *F* (1, 97) = 20.80, *p <* .001, *η*^*2*^ *p* = .18. We also found a significant group by condition interaction, *F* (2, 97) = 17.18, *p <* .001, *η*^*2*^ *p* = .15, with subsequent Bonferroni adjusted (*p =* .008) post hoc tests revealing that non-depressed participants were more effective at generating solutions compared to depressed participants for the baseline, *t*(97) = 5.72, *p <* .001, *d =* 1.14, resolved, *t*(97) = 11.20, *p <* .001, *d =* 2.27, and unresolved conditions, *t*(97) = 12.26, *p <* .001, *d =* 2.24.

Subsequent pairwise analysis revealed that depressed participants were more effective at generating solutions in the baseline than the resolved, *t*(44) = 5.72, *p <* .001, *d =* 1.11, and unresolved conditions, *t*(44) = 8.46, *p <* .001, *d =* 1.36. There were no differences, however, between effectiveness ratings in the resolved and unresolved conditions, *t*(44) = 1.56, *p =* .06, *d =* .31. For the non-depressed participants we found that there were no differences between effectiveness ratings in the baseline and resolved, *t*(53) = .22, *p =* .42, *d =* .03, and unresolved conditions, *t*(53) = .55, *p =* .29, *d =* .08. Furthermore, there were also no differences between the effectiveness ratings in the resolved and unresolved conditions, *t*(53) = .25, *p =* .40, *d =* .04.

### Memory accuracy for consequences

In order to assess recall accuracy for the consequences generated, a 2 (group: depressed vs. control) x 3 (condition: resolved vs. unresolved vs. baseline) mixed design ANOVA was conducted. There were no main effects of condition, *F* (1, 97) = .23, *p =* .79, *η*^*2*^ *p* = .002. A main effect of group, however, was found with non-depressed participants recalling more consequences overall than depressed participants, *F* (1, 97) = 11.96, *p <* .001, *η*^*2*^ *p* = .11. We also found a significant group by condition interaction, *F* (1, 97) = 9.72, *p <* .001, *η*^*2*^ *p* = .09, with the depressed group recalling significantly more unresolved than resolved, *t*(44) = 2.24, *p* = .02, *d* = .58, and baseline consequences, *t*(44) = 1.94, *p* = .03, *d* = .46. There was no difference, however, between their recall of resolved and baseline consequences, *t*(44) = .62, *p =* .27, *d* = .12. Conversely, the non-depressed group recalled significantly more resolved than baseline, *t*(53) = 1.86, *p* = .03, *d* = .35, and, unresolved consequences, *t*(53) = 3.71, *p* < .001, *d* = .71. Non-depressed participants also recalled significantly more baseline than unresolved consequences, *t*(53) = 2.03, *p* = .02, *d* = .37.

Subsequent analysis also found that the non-depressed group recalled significantly more resolved, *t*(97) = 4.57, *p* < .001, *d* = .91, and baseline consequences compared to the depressed participants, *t*(97) = 2.45, *p* = .01, *d* = .49. There was no difference, however, between depressed and non-depressed groups in their recall of unresolved consequences, *t*(97) = 1.68, *p* = .05, *d* = .34. See Table 2.

**Table 2:** Showing group differences in problem-solving.

	Depressed Group Mean (SD)		Non-Depressed Group Mean (SD)
**Rel. Means *(Resolved)***		3.13 (1.27)	5.09 (1.03)
**Rel. Means *(Unresolved)***		2.78 (1.06)	5.08 (1.04)
**Rel. Means *(Baseline)***		4.23 (1.22)	5.36 (1.24)
**Effectiveness *(Resolved)***		2.90 (.94)	5.03 (.94)
**Effectiveness *(Unresolved)***		2.58 (1.09)	4.99 (.87)
**Effectiveness *(Baseline)***		3.99 (1.02)	5.06 (.85)
**Consequences *(Resolved)***		47.78 (29.11)	72.22 (24.12)
**Consequences *(Unresolved)***		63.33 (24.77)	55.09 (23.98)
**Consequences *(Baseline)***		51.11 (28.18)	63.89 (23.63)

Rel. Means = Relevant Means; Effectiveness = Effectiveness ratings for proposed solutions; Consequences = Recall accuracy for consequences generated.

### The relationship between depression, rumination and intrapersonal problem-solving

Pearson correlations were conducted separately for the depressed and non-depressed control group in order to explore whether there was a relationship between depression, rumination and intrapersonal problem-solving (relevant- means and effectiveness ratings). For the depressed group it was found that there were significant negative relationships between depression scores and intrapersonal problem-solving, rumination scores and intrapersonal problem-solving and a positive relationship between depression and rumination scores. See Table 3 for a full breakdown of the relationships. Our analysis, however, failed to find any significant correlations between depression scores and intrapersonal problem-solving, rumination scores and intrapersonal problem-solving or between depression and rumination scores for the non-depressed control group; all tests p > .05.

**Table 3:** Pearson correlation coefficients for depressed participants between the percentage of consequences recalled consequences that were recalled by participants in the consequence recall phase., problem-solving and self-reported depression and rumination scores.

	Depression	Rumination	Steps-R	Steps- UR	Steps-B	Effect-R	Effect-UR	Effect-B	Cons-R	Cons-UR	Cons-B
**Depression**	---	.682***	-.451**	-.585***	-.436**	-.494***	-.368*	-.395**	-.559***	.632***	-.371***
**Rumination**	.682***	---	-.336*	-.440**	-.386**	-.400**	-.367*	-.432**	-.320*	.502***	-.417**
**Steps-R**	-.451**	-.336*	---	.217	.232	.249	.440**	.225	.234	.093	-1.84
**Steps-UR**	-.585***	-.440**	.217	---	.168	.137	.450**	.235	.113	.343*	-.210
**Steps-B**	-.436**	-.386**	.232	.168	---	.303*	.054	.084	.157	.167	-.339*
**Effect-R**	-.494***	-.400**	.249	.137	.303*	---	.069	.153	.294	.148	-.210
**Effect-UR**	-.368*	-.367*	.440**	.450**	.054	.069	---	.437**	.359*	.095	-.229
**Effect-B**	-.395**	-.432**	.225	.235	.084	.153	.437**	---	.386**	.353*	-.331*
**Cons-R**	-.559***	-.320*	.093	.343*	.167	.148	.095	.353*	---	-.490***	.211
**Cons-UR**	.632***	.502***	-1.84	-.210	-.339*	-.210	-.229	-.331*	-.490***	---	-.266
**Cons-B**	-.371***	-.417**	.234	.113	.157	.294	.359*	.386**	.211	-.266	---

* P < 0.05

** p < 0.01

*** p < 0.001.

### Regression analyses

Given that we found significant correlations between depression and rumination scores in the depressed group, two-stage hierarchical multiple regression analyses were conducted with intrapersonal problem-solving measures and memory recall of consequences as the dependent measures. Depression scores were entered at stage one of the regressions and rumination scores were entered at stage 2. See Table 4 for the regression analysis results.

**Table 4:** Hierarchical regression analyses results.

		F	df	*p*	R_2_	∆R_2_	Beta	t
**Baseline- Relevant**	***MODEL 1***	10.11	(2, 42)	.003	.19	.17	.44	3.18**
**Means**	Depression							
	***MODEL 2***	5.42	(2, 42)	.008	.21	.17		
	Depression & Rumination							
	**Change in R**_**2**_	**.78**	**(1, 42)**	**.38**				
**Resolved- Relevant**	***MODEL 1***	10.99	(2, 42)	.002	.20	.19	.45	3.32**
**Means**	Depression							
	***MODEL 2***	5.42	(2, 42)	.008	.21	.17		
	Depression & Rumination							
	**Change in R**_**2**_	**.08**	**(1, 42)**	**.78**				
**Unresolved-**	***MODEL 1***	22.36	(2, 42)	< .001	.34	.33	.59	4.73***
**Relevant Means**	Depression							
	***MODEL 2***	11.07	(2, 42)	< .001	.35	.31		
	Depression & Rumination							
	**Change in R**_**2**_	**.20**	**(1, 42)**	**.66**				
**Baseline-**	***MODEL 1***	7.96	(2, 42)	.007	.16	.14	.39	2.82**
**Effectiveness**	Depression							
	***MODEL 2***	5.43	(2, 42)	.008	.21	.17		
	Depression & Rumination							
	**Change in R**_**2**_	**2.61**	**(1, 42)**	**.11**				
**Resolved-**	***MODEL 1***	13.88	(2, 42)	< .001	.24	.23	.49	3.73***
**Effectiveness**	Depression							
	***MODEL 2***	7.06	(2, 42)	.002	.25	.22		
	Depression & Rumination							
	**Change in R**_**2**_	.42	**(1, 42)**	.52				
**Unresolved-**	***MODEL 1***	6.72	(2, 42)	.01	.14	.12	.37	2.59*
**Effectiveness**	Depression							
	***MODEL 2***	4.02	(2, 42)	.03	.16	.12		
	Depression & Rumination							
	**Change in R**_**2**_	**1.27**	**(1, 42)**	**.27**				
**Baseline- Consequences**	***MODEL 1***	6.89	(2, 42)	.01	.14	.12	.37	2.62*
	Depression							
	***MODEL 2***	4.85	(2, 42)	.01	.19	.15		
	Depression & Rumination							
	**Change in R**_**2**_	**2.60**	**(1, 42)**	**.11**				
**Resolved- Consequences**	***MODEL 1***	19.57	(2, 42)	< .001	.31	.30	.56	4.42***
	Depression							
	***MODEL 2***	9.88	(2, 42)	< .001	.32	.29		
	Depression & Rumination							
	**Change in R**_**2**_	**.44**	**(1, 42)**	**.51**				
**Unresolved-Consequences**	***MODEL 1***	28.60	(2, 42)	< .001	.40	.39	.63	5.35***
	Depression							
	***MODEL 2***	14.53	(2, 42)	< .001	.41	.38		
	Depression & Rumination							
	**Change in R**_**2**_	**.68**	**(1, 42)**	**.42**				

The regression analysis for relevant-means found that in the baseline (i.e., when no consequences were generated), resolved and unresolved conditions, depression was significantly associated with the number of relevant means and accounted for 17%, 19% and 33% of variance, respectively. Rumination, however, did not significantly predict the number of relevant means, over and above the effects of depression, across all three conditions. The analysis for the effectiveness of the proposed solutions found that in the baseline, resolved and unresolved conditions, depression significantly predicted the effectiveness of the proposed solutions, explaining 14%, 23% and 12% of the variance, respectively. Rumination, however, did not significantly predict the effectiveness of solutions, over and above the effects of depression, across all three conditions. Finally, regression analysis for the consequences that were recalled also showed a similar pattern, with depression predicting the number of consequences recalled across baseline, resolved and unresolved conditions, explaining 12%, 30%, and 39% of the variance, respectively. However, rumination did not significantly predict the number of consequences recalled over and above the effects of depression.

## Discussion

The aim of the current study was to investigate the impact of depression on intrapersonal problem-solving when participants were primed to engage in negative versus positive future episodic simulations related to the problems. We predicted that depressed individuals would generate fewer relevant means and less effective solutions in the baseline condition where no consequences were generated compared to non-depressed controls. The findings supported our prediction and are consistent with numerous studies that have found that depressed individuals are impaired at social problem-solving [1–4, 8]. Given that this study focused on intrapersonal problems whereas previous research has almost exclusively examined interpersonal problem-solving, these findings extend previous work and suggest that deficits in problem-solving in depression are not a function of interpersonal problems per se but may represent a more global deficit in problem-solving ability. Further support for negative moods having a more general impairment on problem-solving comes from research which has found that positive compared to negative moods facilitate performance on a range of measures of creative problem-solving ability [77–79]. As positive moods are more likely to involve using simple heuristics and loose processing, they increase the likelihood of generating creative solutions [80–82].

We also predicted that depressed individuals would generate fewer relevant-means and less effective solutions for problems following the generation of unresolved consequences compared to non-depressed participants. This prediction was supported by our results and is consistent with findings that show depression is associated with impaired problem-solving in the unresolved consequence condition [1, 2]. We also found that depressed individuals were less effective at problem-solving following the generation of unresolved consequences, compared to the baseline condition, which supports our recent findings [1] and suggests that generating unresolved consequences may act to further exacerbate existing problem-solving deficits in depression.

One reason for these findings may be that encouraging depressed individuals to think about the consequences of a problem remaining unresolved results in them focusing on these consequences, believing them more likely to occur, which subsequently leads to the belief that the problem is overwhelming and unsolvable. This argument is supported by research which has found that depressed compared to non-depressed individuals show a greater belief in the likelihood of negative future-events occurring [83, 84], anticipate fewer positive experiences in the future [85] and show pessimistic attitudes and have low expectations about the future [86–88]. Thus, encouraging depressed participants to think about the consequences of problem remaining unresolved may trigger pessimistic future event schemas which lead to the faulty belief that the problem is unsolvable.

Alternatively, it is possible that generating unresolved consequences leads to depressed individuals focusing their attention on the unresolved consequences during problem-solving which subsequently impairs their ability to solve the problems effectively. This explanation is consistent with research which suggests that depressed individuals allocate more attention to negative rather than positive or neutral material [89, 90] and exhibit difficulties disengaging from it [91–93]. Given that unresolved consequences are more likely to be negative in content, it is possible that once depressed participants generated them, they focused their attention on these consequences and found it difficult to disengage from thinking about them, which in turn, had a detrimental impact on problem-solving. Further support for difficulties in disengagement is revealed by our findings showing that depressed individuals recalled significantly more unresolved than resolved or baseline consequences (i.e., problems where consequences were generated but participants did not engage in problem-solving) whilst non-depressed individuals recalled significantly more resolved than unresolved or baseline consequences. Given that resolved consequences were more positive in content whilst unresolved consequences were largely negative In order to investigate the valence of the consequences generated, we conducted a 2 (group; depressed vs. non-depressed control) x 2 (Consequences; resolved vs. unresolved) x 3 (Valence; positive vs. negative & neutral) mixed design ANOVA. Our analysis revealed that resolved consequences were positive rather than negative in content whilst unresolved consequences were more negative than positive in content; *F* (1, 97) = 6359.90, *p <* .001, *η*^*2*^ *p* = .97., these findings are consistent with the mood congruency literature which has reported that depressed and dysphoric individuals show enhanced memory for negative material whilst non-depressed participants show enhanced memory for positive material [94, 95]. Moreover, the findings are also consistent with our previous findings [1, 2].

Interestingly, we also found that depressed individuals demonstrated impaired intrapersonal problem-solving following the generation of resolved consequences compared to non-depressed participants and the baseline condition. These findings are contrary to previous findings [1, 2] which found that generating resolved consequences enhanced problem-solving ability in depressed and dysphoric individuals and suggest that problem-solving deficits in depression may depend on the nature of the problem being resolved. In the present study we used intrapersonal problems whilst previous studies have focused on interpersonal problem-solving. Given that intrapersonal problems are self-focused and interpersonal problems involve others, it is possible that these two forms of problem-solving rely on different mechanisms.

Intrapersonal problem-solving, for example, is more dependent on one’s perception of self. As a result, it is possible that generating the consequences of the problem being resolved creates a larger discrepancy between one’s *actual* self (i.e., faced with a problem) vs. their *ideal* self (problem being resolved). This discrepancy between *actual* and *ideal* self may make depressed individuals more vulnerable to experience further negative emotions, such as sadness, disappointment and dissatisfaction. This, in turn, may affect both their emotional and motivational state and subsequently impair problem-solving ability. This explanation is consistent with research that has found that depressed individuals demonstrate higher levels of self-discrepancy than non-depressed individuals [96, 97]. It has also been found that whilst depressed individuals recalling a positive memory concordant with their current self-improves their mood, recalling positive self-discrepant memories do not [62]. Moreover, research suggests that the discrepancy between *actual* self vs. *ideal* self may serve as a cognitive vulnerability mechanism that contributes to the onset and maintenance of depression [98].

Related to this, given that the intrapersonal problems presented in the current study (*i*.*e*., w*ishing to lose weight*, *having to present a presentation in front of others*, *etc*.) are problems inherently focused on self and a distorted perception of self is central to the aetiology of depression [99], it is possible that these problems may be the type of intrapersonal problems that depressed individuals experience in daily life and have difficulties with resolving. Furthermore, it is also possible that these unresolved problems may be a contributory factor in the onset and maintenance of their depression. If this is the case, then generating positive consequences of the problem being resolved may be detrimental for depressed individuals and further exacerbate existing discrepancies between *actual* self and *ideal* self subsequently impairing intrapersonal problem-solving. This conclusion is supported by research which has found that depressed individuals generate more negative evaluations of the self. For example, it has been found that depressed individuals attribute more negative traits and characteristics to themselves [100] and endorse more negative and fewer positive words to themselves (i.e., self-referent) compared to non-depressed participants [101].

Our study also found that depressed individuals show greater deficits in problem-solving following the generation of both resolved and unresolved consequences compared to a baseline condition where no consequences were generated. These findings are contrary to previous findings [1, 2] and suggest that future episodic simulation, regardless of the content of one’s thoughts may impede intrapersonal problem-solving. One reason for this may be that generating consequences for intrapersonal problems may create a negative problem orientation in depressed individuals which then impacts on their ability to solve the problem. Numerous studies have found that depression is associated with a negative problem orientation [13, 19–21]. The way in which negative problem orientation arises, however, may differ depending on the consequences generated. For example, unresolved consequences are more likely congruent with depressed mood whilst resolved consequences produce a larger discrepancy between *actual* vs. *ideal* self, both of which maintain and exacerbate an existing negative problem orientation. Future research may wish to investigate the role of negative problem-orientation in depression and its impact on future episodic simulation and intrapersonal problem-solving.

Interestingly, we found that rumination did not predict impaired social problem-solving in depression when controlling for depression in the baseline, resolved and unresolved conditions. These results are contrary to previous findings that rumination predicts social problem-solving deficits in depression individuals in the unresolved condition [1, 2]. The findings are surprising given the self-focused nature of both intrapersonal problems and rumination and point to the possibility that rumination may play a different role in inter- and intrapersonal problem-solving. As mentioned earlier, given that intrapersonal problems are more closely related to one’s self-perception, and a negative self-perception is a central feature in depression [102–104] it is possible that these problems may have been personally experienced in daily life and continue to remain unresolved, possibly contributing to the maintenance of depressed mood.

These ongoing unresolved problems may also foster a sense of hopelessness. Hopelessness involves holding negative expectations about one’s future [99]. Research has found that people that hold these negative expectations cannot solve their problems or reach their goals [105]. Furthermore, depressed individuals often report high levels of hopelessness [106]. Thus, depressed individuals may develop a sense of hopelessness regarding intrapersonal problems, believing them to be unsolvable which then diminishes ruminative thinking over these problems. This is in line with research which suggests that although rumination is a repetitive conscious process that involves focusing on one’s recent problems or failures, people who ruminate often believe that it is a way to help solve their problems [107]. However, if the problem is perceived as unsolvable, then depressed individuals may be less inclined to ruminate on these problems, and instead focus on ruminating over more recent or immediate problems. Consistent with this argument, it has been found that problem-solving difficulties and increased hopelessness are associated with avoidant coping [108–110]. Therefore, it is possible that if a depressed individual feels unable to resolve their problem, they may avoid the problem altogether, thereby reducing rumination. The fact that rumination predicts social problem deficits in interpersonal but not intrapersonal problem-solving is intriguing and points to the possibility that it may have differing effects on different types of problems. Future research is needed to distinguish the effects of rumination on inter- and intrapersonal problem-solving.

We also found that non-depressed participants showed no significant differences in problem-solving ability across baseline, resolved and unresolved conditions. These findings are consistent with previous research [1]. One of the reasons why problem-solving performance was not influenced by the generation of the resolved and unresolved consequences may be due to inhibition. Inhibition involves preventing unwanted or distracting information from coming to mind to reduce its impact on ongoing information processing [111, 112]. In the present task, once participants have generated the consequences of the problem being resolved or remaining unresolved, they must subsequently inhibit these consequences in order to solve the problem. Difficulties inhibiting previously generated consequences may subsequently affect their ability to think clearly about the steps needed to solve a problem, thus resulting in impaired social problem-solving. Research has found that non-depressed participants have good inhibitory control compared to non-depressed participants [1, 113]. As non-depressed participants have good inhibitory control, it is possible that when asked to solve the problem they were able to prevent the consequences from coming to mind, and instead focused on solving the problem.

### Limitations

It is important to mention that one limitation of the current study is that we did not assess whether participants had personally experienced any of the problems presented and whether these problems were resolved or currently ongoing. This is an important factor given that problem-solving is associated with the ability to retrieve specific memories of past experiences [23, 51, 114] which may act as simulations for generating hypothetical solutions to problems [46]. For example, individuals with depression recall significantly fewer specific memories and generate fewer effective solutions to hypothetical problems compared to people with higher specificity [23, 115, 116], suggesting that autobiographical memory specificity may be beneficial to problem-solving. Thus, having information on whether participants had personally experienced any of the problems, and the level of specificity of the memory that was retrieved, may provide more insight into the relationship between future episodic simulations, intrapersonal problem-solving and depression.

It is important to mention that whilst our depressed participants met the diagnostic criteria for clinical depression, the majority of our sample also suffered from other comorbid conditions, such as anxiety disorders, eating disorders and social phobia. Given that research has found that social problem-solving is impaired in other clinical conditions, such as social anxiety and eating disorders [117, 118], it is possible that our findings can be attributed to the presence of these comorbid disorders rather than depression per se. However, it is important to mention that our findings that depressed participants are impaired at social problem-solving are consistent with previous research [6, 19, 23]. Furthermore, our depressed sample is also representative of the general clinical population which often has high rates of comorbidity, with depression often accompanied by other mental disorders [119]. Nevertheless, future research should investigate future episodic simulations and intrapersonal problem-solving in ‘purely’ depressed participants, in the absence of any comorbid disorders.

Another limitation is that whilst our findings show impaired problem-solving deficits in depressed individuals for intrapersonal problems in the resolved condition, no direct comparison was made between interpersonal and intrapersonal problem-solving in depression so we cannot sure that there are inherent differences between these problems and that intrapersonal problem-solving is more difficult for depressed individuals. Future research should investigate both forms of problem-solving in depression in order to more strongly support the dissociation between future episodic simulation and inter- and intrapersonal problem-solving in depression. Furthermore, future research should also test the role of self-discrepancy on problem-solving by using a measure of self-discrepant thinking to determine how it relates to intrapersonal problem-solving ability in depression.

A final limitation of the present study is that as our depressed sample consisted of only 45 participants, we were unable to run regression analyses to test the interaction between problem condition (baseline, resolved, unresolved) with depression and rumination scores due to insufficient power. Future research should use a larger sample size and ensure that it is sufficiently powered to run these multiple regression analyses.

### Clinical implications

The current findings have implications for problem-solving training. For example, previous efforts to increase the effectiveness of problem-solving skills have been developed in response to research largely dominated by interpersonal problem-solving [15, 17, 120]. Whilst some research suggests that problem-solving therapies have been effective in depression [121–123], other studies have failed to find significant effects ([124] see [125] for a review on the mixed effects). Indeed Ehring et al. [16] has identified that social problem-solving is one area where translational research from mechanisms into clinical practice is currently lacking.

Recent findings suggest that future episodic simulation may aid social problem-solving in depressed individuals. For example, thinking about the consequences of a problem being resolved has been found to improve interpersonal problem-solving in depression [1]. Our current findings, however, suggest that for intrapersonal problems, generating consequences may impede or hinder depressed individuals’ efforts to solve problems and may be counterproductive. Thinking about an intrapersonal problem being resolved may be more likely to induce self-discrepant thinking in depressed individuals if they currently face similar problems that are unsolvable. Self-discrepant thinking can lead to greater negative self-reflection and rumination [28] which would impair problem-solving. Similarly thinking about problems being unresolved may also enhance rumination about deficits in the self, making problem-solving more difficult. These findings are in line with the paradoxical effect of positive autobiographical memory retrieval producing poorer mood in depressed individuals if the memories are discrepant with their current view of the self [62].

Our findings instead suggest that different intervention strategies may be needed for different real-life problem-solving contexts. For example, for interpersonal problems, encouraging depressed individuals to ‘*zoom out’* from the problem and consider the wider second order consequences of the problem being resolved may enhance problem solving ability, whilst for intrapersonal problems, depressed participants should be encouraged to ‘*zoom in*’ and focus on the problem itself and how to solve the problem without thinking about the wider implications of the problem being resolved or remaining unresolved.

## Conclusion

To conclude, this study is the first to investigate intrapersonal problem-solving and future episodic simulation in depression. Our results have found that depressed individuals, compared to non-depressed individuals generate fewer-relevant means and less effective solutions to problems in the baseline, resolved and unresolved conditions. These findings are contrary to our previous findings [1, 2] which showed that generating positive consequences of a problem being resolved improved social problem-solving ability in depressed participants. Taken together, our findings suggest that a more nuanced approach to future thinking and problem-solving in depression is needed across different real-life problem-solving contexts.

## Supporting information

S1 Table (DOCX)

## References

[1] Noreen S, Dritschel B. In the here and now: Future thinking and social problem-solving in depression. Manelis A, editor. PLOS ONE. 2022 Jun 30;17(6):e0270661. doi: 10.1371/journal.pone.0270661 35771846 PMC9246169

[2] Noreen S, Whyte KE, Dritschel B. Investigating the role of future thinking in social problem-solving. Journal of Behavior Therapy and Experimental Psychiatry. 2015 Mar;46:78–84. doi: 10.1016/j.jbtep.2014.08.004 25252189

[3] Haaga DAF, Fine JA, Terrill DR, Stewart BL, Beck AT. Social problem-solving deficits, dependency, and depressive symptoms. Cognitive Therapy and Research. 1995 Apr;19(2):147–58.

[4] Nezu AM. A problem-solving formulation of depression: A literature review and proposal of a pluralistic model. Clinical Psychology Review. 1987 Jan;7(2):121–44.

[5] Beck AT. Cognitive therapy and the emotional disorders. London: Penguin; 1991.

[6] D’Zurilla TJ, Sheedy CF. Relation between social problem-solving ability and subsequent level of psychological stress in college students. Journal of Personality and Social Psychology. 1991;61(5):841–6. doi: 10.1037//0022-3514.61.5.841 1753336

[7] Heppner PP, Krauskopf CJ. An Information-Processing Approach to Personal Problem-solving. The Counseling Psychologist. 1987 Jul;15(3):371–447.

[8] Marx EM, Williams JM, Claridge GC. Depression and social problem-solving. Journal of Abnormal Psychology. 1992 Feb;101(1):78–86. doi: 10.1037//0021-843x.101.1.78 1537977

[9] Asarnow JR, Carlson GA, Guthrie D. Coping strategies, self-perceptions, hopelessness, and perceived family environments in depressed and suicidal children. Journal of Consulting and Clinical Psychology. 1987;55(3):361–6 doi: 10.1037//0022-006x.55.3.361 3597949

[10] Fremouw W, Callahan T, Kashden J. Adolescent suicidal risk: psychological, problem-solving, and environmental factors. Suicide Life Threat Behav. 1993;23(1):46–54. 8475532

[11] Rotheram-Borus MJ. Adolescents’ reference-group choices, self-esteem, and adjustment. Journal of Personality and Social Psychology. 1990;59(5):1075–81. doi: 10.1037//0022-3514.59.5.1075 2266482

[12] Speckens AEM, Hawton K. Social Problem-solving in Adolescents with Suicidal Behavior: A Systematic Review. Suicide and Life-Threatening Behavior. 2005 Aug;35(4):365–87. doi: 10.1521/suli.2005.35.4.365 16178693

[13] Becker-Weidman EG, Jacobs RH, Reinecke MA, Silva SG, March JS. Social Problem-Solving among Adolescents Treated for Depression. Behaviour research and therapy [Internet]. 2010 Jan 1;48(1):11–8. Available from: https://www.ncbi.nlm.nih.gov/pmc/articles/PMC2812620/ doi: 10.1016/j.brat.2009.08.006 19775677 PMC2812620

[14] Chang EC, D’zurilla TJ, Sanna LJ, editors. Social problem-solving: theory, research, and training. Washington, Dc: American Psychological Association; 2004.

[15] D’Zurilla TJ, Nezu AM. Problem-solving Therapy. Springer Publishing Company; 1999.

[16] Ehring T, Limburg K, Kunze AE, Wittekind CE, Werner GG, Wolkenstein L, et al. (When and how) does basic research in clinical psychology lead to more effective psychological treatment for mental disorders? Clinical Psychology Review [Internet]. 2022 Jul 1 [cited 2022 Jun 8];95:102163. doi: 10.1016/j.cpr.2022.102163 35660924

[17] D’Zurilla TJ. Problem-Solving Therapy: A Social Competence Approach to Clinical Intervention. New York: Springer.1986.

[18] Ben-Zur H. Coping styles and affect. International Journal of Stress Management. 2009 May;16(2):87–101.

[19] Priester MJ, Clum GA. Perceived problem-solving ability as a predictor of depression, hopelessness, and suicide ideation in a college population. Journal of Counseling Psychology. 1993;40(1):79–85.

[20] Anderson R.J., Goddard L. & Powell J.H. Social Problem-Solving and Depressive Symptom Vulnerability: The Importance of Real-Life Problem-Solving Performance. Cogn Ther Res 35, 48–56 (2011). 10.1007/s10608-009-9286-2

[21] Sun RCF, Shek DTL. Life Satisfaction, Positive Youth Development, and Problem Behaviour Among Chinese Adolescents in Hong Kong. Social Indicators Research. 2010;95(3):455–74. doi: 10.1007/s11205-009-9531-9 20062815 PMC2801834

[22] Reinecke MA, DuBois DL, Schultz TM. Social Problem-solving, Mood, and Suicidality Among Inpatient Adolescents. Cognitive Therapy and Research. 2001: 25:743–756.

[23] Goddard L, Dritschel B, Burton A. Role of autobiographical memory in social problem-solving and depression. Journal of Abnormal Psychology. 1996 Nov;105(4):609–16. doi: 10.1037//0021-843x.105.4.609 8952194

[24] Watkins E, Baracaia S. Rumination and social problem-solving in depression. Behaviour Research and Therapy. 2002 Oct;40(10):1179–89. doi: 10.1016/s0005-7967(01)00098-5 12375726

[25] Watkins E, Moulds M. Distinct modes of ruminative self-focus: Impact of abstract versus concrete rumination on problem-solving in depression. Emotion. 2005;5(3):319–28. doi: 10.1037/1528-3542.5.3.319 16187867

[26] Goddard RD. Collective efficacy: A neglected construct in the study of schools and student achievement. Journal of Educational Psychology. 2001;93(3):467–76.

[27] Nolen-Hoeksema S. Responses to depression and their effects on the duration of depressive episodes. Journal of Abnormal Psychology. 1991;100(4):569–82. doi: 10.1037//0021-843x.100.4.569 1757671

[28] Watkins ER. Constructive and unconstructive repetitive thought. Psychological Bulletin. 2008 Mar;134(2):163–206. doi: 10.1037/0033-2909.134.2.163 18298268 PMC2672052

[29] Nolen-Hoeksema S. The role of rumination in depressive disorders and mixed anxiety/depressive symptoms. Journal of Abnormal Psychology. 2000;109(3):504–11. 11016119

[30] Watkins ER, Roberts H. Reflecting on rumination: Consequences, causes, mechanisms and treatment of rumination. Behaviour Research and Therapy. 2020 Apr;127:103573. doi: 10.1016/j.brat.2020.103573 32087393

[31] Lyubomirsky S, Tucker KL, Caldwell ND, Berg K. Why ruminators are poor problem solvers: Clues from the phenomenology of dysphoric rumination. Journal of Personality and Social Psychology. 1999;77(5):1041–60. doi: 10.1037//0022-3514.77.5.1041 10573879

[32] Donaldson C, lam D. Rumination, mood and social problem-solving in major depression. Psychological Medicine. 2004 Oct;34(7):1309–18. doi: 10.1017/s0033291704001904 15697057

[33] O’Mahen HA, Boyd A, Gashe C. Rumination decreases parental problem-solving effectiveness in dysphoric postnatal mothers. Journal of Behavior Therapy and Experimental Psychiatry. 2015 Jun;47:18–24. doi: 10.1016/j.jbtep.2014.09.007 25462598

[34] Szpunar KK, Spreng RN, Schacter DL. A taxonomy of prospection: introducing an organizational framework for future-oriented cognition. *Proc Natl Acad Sci U S A*. 2014;111(52):18414–18421. doi: 10.1073/pnas.1417144111 25416592 PMC4284580

[35] Suddendorf T, Corballis MC. The evolution of foresight: What is mental time travel, and is it unique to humans? Behavioral and Brain Sciences [Internet]. 2007;30(03). Available from: https://espace.library.uq.edu.au/view/UQ:135952/Suddendorf-04122006.pdf doi: 10.1017/S0140525X07001975 17963565

[36] Tulving E. (2005). Episodic Memory and Autonoesis: Uniquely Human? In Terrace H. S. & Metcalfe J. (Eds.), The missing link in cognition: Origins of self-reflective consciousness (pp. 3–56). Oxford University Press. 10.1093/acprof:oso/9780195161564.003.0001

[37] Klinger E, Cox WM. Dimensions of Thought Flow in Everyday Life. Imagination, Cognition and Personality [Internet]. 1987 Oct [cited 2021 Feb 19];7(2):105–28.

[38] Dalgleish T, Werner-Seidler A. Disruptions in autobiographical memory processing in depression and the emergence of memory therapeutics. Trends in Cognitive Sciences. 2014 Nov;18(11):596–604. doi: 10.1016/j.tics.2014.06.010 25060510

[39] D’Argembeau A, Ortoleva C, Jumentier S, Van der Linden M. Component processes underlying future thinking. Memory & Cognition. 2010 Sep;38(6):809–19. doi: 10.3758/MC.38.6.809 20852243

[40] D’Argembeau A, Renaud O, Van der Linden M. Frequency, characteristics and functions of future-oriented thoughts in daily life. Applied Cognitive Psychology. 2011 Jan;25(1):96–103.

[41] Madore KP, Schacter DL. An episodic specificity induction enhances means-end problem-solving in young and older adults. Psychology and Aging. 2014;29(4):913–24. doi: 10.1037/a0038209 25365688 PMC4268420

[42] Schacter DL, Madore KP. Remembering the past and imagining the future: Identifying and enhancing the contribution of episodic memory. Schacter DL, Welker M, editors. Memory Studies. 2016 Jun 30;9(3):245–55. doi: 10.1177/1750698016645230 28163775 PMC5289412

[43] Sheldon S, McAndrews MP, Moscovitch M. Episodic memory processes mediated by the medial temporal lobes contribute to open-ended problem-solving. Neuropsychologia. 2011 Jul;49(9):2439–47. doi: 10.1016/j.neuropsychologia.2011.04.021 21550352

[44] Schacter DL, Addis DR, Buckner RL. Remembering the past to imagine the future: the prospective brain. Nature Reviews Neuroscience. 2007 Sep;8(9):657–61. doi: 10.1038/nrn2213 17700624

[45] Schacter DL, Addis DR, Buckner RL. Episodic simulation of future events: concepts, data, and applications. Ann N Y Acad Sci. 2008;1124:39–60. doi: 10.1196/annals.1440.001 18400923

[46] Beaman A, Pushkar D, Etezadi S, Bye D, Conway M. Autobiographical Memory Specificity Predicts Social Problem-Solving Ability in Old and Young Adults. Quarterly Journal of Experimental Psychology. 2007 Sep;60(9):1275–88. doi: 10.1080/17470210600943450 17676558

[47] Williams JM, Barnhofer T, Crane C. Autobiographical memory specificity and emotional disorder. Psychol Bull. 2007;133(1):122–148. doi: 10.1037/0033-2909.133.1.122 17201573 PMC2834574

[48] MacLeod AK, Byrne A. Anxiety, depression, and the anticipation of future positive and negative experiences. Journal of Abnormal Psychology. 1996 May;105(2):286–9. doi: 10.1037//0021-843x.105.2.286 8723011

[49] Miranda R, Mennin DS. Depression, Generalized Anxiety Disorder, and Certainty in Pessimistic Predictions about the Future. Cognitive Therapy and Research. 2007 Feb 21;31(1):71–82.

[50] Miloyan B, Pachana NA, Suddendorf T. Future-Oriented Thought Patterns Associated With Anxiety and Depression in Later Life: The Intriguing Prospects of Prospection. The Gerontologist. 2016 Feb 13;57(4):gnv695. doi: 10.1093/geront/gnv695 26874188 PMC5881767

[51] Lavender A, Watkins E. Rumination and future thinking in depression. British Journal of Clinical Psychology. 2004 Jun;43(2):129–42. doi: 10.1348/014466504323088015 15169614

[52] Dennis AA, Astell A, Dritschel B. The effects of imagery on problem-solving ability and autobiographical memory. J Behav Ther Exp Psychiatry. 2012;43 Suppl 1:S4–S11. doi: 10.1016/j.jbtep.2011.06.002 23200430

[53] Barber BK. Positive interpersonal and intrapersonal functioning: An assessment of measures among adolescents. In: Moore KA, Lippman L. What do children need to flourish?: conceptualizing and measuring indicators of positive development. New York: Springer; 2005. P147–161.

[54] Finkel EJ, Vohs KD. Introduction. Self and Relationships. In: Vohs KD, Finkel EJ. Self and relationships: connecting intrapersonal and interpersonal processes. New York: Guilford Press; 2006: pp. 1–9.

[55] Alcaro A, Panksepp J, Witczak J, Hayes DJ, Northoff G. Is subcortical-cortical midline activity in depression mediated by glutamate and GABA? A cross-species translational approach. Neurosci Biobehav Rev. 2010;34(4):592–605. doi: 10.1016/j.neubiorev.2009.11.023 19958790

[56] Hoffmann F, Banzhaf C, Kanske P, Bermpohl F, Singer T. Where the depressed mind wanders: Self-generated thought patterns as assessed through experience sampling as a state marker of depression. *J Affect Disord*. 2016;198:127–134. doi: 10.1016/j.jad.2016.03.005 27015160

[57] Alloy LB, Abramson LY, Francis EL. Do negative cognitive styles confer vulnerability to depression? Current Directions in Psychological Science.1999;8(4):128–132.

[58] Ji JL, Grafton B, MacLeod C. Referential focus moderates depression-linked attentional avoidance of positive information. *Behav Res Ther*. 2017;93:47–54. doi: 10.1016/j.brat.2017.03.004 28384508 PMC5408905

[59] Beck AT. Cognitive Therapy and the Emotional Disorders, Chapter 9, “Principles of Cognitive Therapy.” New York, Ny: International Universities Press; 1976.

[60] Abramson LY, Metalsky GI, Alloy LB. Hopelessness depression: A theory-based subtype of depression. *Psychological Review*. 1989; 96(2), 358–372.

[61] Joiner Jr, Thomas E. "A test of the hopelessness theory of depression in youth psychiatric inpatients." *Journal of Clinical Child Psychology* 29.2 (2000): 167–176. doi: 10.1207/S15374424jccp2902_3 10802826

[62] Werner-Seidler A, Tan L, Dalgleish T. The Vicissitudes of Positive Autobiographical Recollection as an Emotion Regulation Strategy in Depression. Clinical Psychological Science. 2016 Aug 20;5(1):26–36.

[63] Szpunar KK. Episodic Future Thought: An Emerging Concept. Perspect Psychol Sci. 2010 Mar;5(2):142–62. doi: 10.1177/1745691610362350 . 26162121

[64] Fergus TA, Valentiner DP, Wu KD, McGrath PB. Examining the symptom level specificity of negative problem orientation in a clinical sample. Cognitive Behaviour Therapy. 2015; 44, 153–161. doi: 10.1080/16506073.2014.987314 25491568

[65] Sheehan DV, Lecrubier Y, Sheehan KH, Amorim P, Janavs J, Weiller E. The Mini-International Neuropsychiatric Interview (M.I.N.I.): the development and validation of a structured diagnostic psychiatric interview for DSM-IV and ICD-10. J Clin Psychiatry. 1998;59 Suppl 20:2 9881538

[66] Beck AT, Steer RA, Brown GK. BDI-II: Beck depression inventory. San Antonio Tex.: Psychological Corp; 1996.

[67] Faul F, Erdfelder E, Lang AG, Buchner A. G*Power 3: A Flexible Statistical Power Analysis Program for the Social, Behavioral, and Biomedical Sciences. Behavior Research Methods. 2007; 39, 175–191. doi: 10.3758/bf03193146 17695343

[68] Sheehan DV, Lecrubier Y, Sheehan KH, Janavs J, Weiller E, Keskiner A, Dunbar CG. The validity of the Mini International Neuropsychiatric Interview (MINI) according to the SCID-P and its reliability. European psychiatry.1997;12(5):232–241.

[69] Lecrubier Y, Sheehan D, Weiller E, Amorim P, Bonora I, Harnett Sheehan K, et al. The Mini International Neuropsychiatric Interview (MINI). A short diagnostic structured interview: reliability and validity according to the CIDI. European Psychiatry. 1997;12(5):224–31.

[70] Beck AT, Steer RA, Carbin MG. Psychometric properties of the Beck Depression Inventory: Twenty-five years of evaluation. Clinical Psychology Review [Internet]. 1988 Jan;8(1):77–100.

[71] Spielberger CD, Gorsuch RL, Lushene R, Vagg PR, Jacobs GA. *Manual for the State-Trait Anxiety Inventory*. Palo Alto, CA: Consulting Psychologists Press. 1983.

[72] Kabacoff RI, Segal DL, Hersen M, Van Hasselt VB. Psychometric properties and diagnostic utility of the Beck Anxiety Inventory and the state-trait anxiety inventory with older adult psychiatric outpatients. Journal of Anxiety Disorders. 1997 Jan;11(1):33–47. doi: 10.1016/s0887-6185(96)00033-3 9131880

[73] Oei TPS, Evans L, Crook GM. Utility and validity of the STAI with anxiety disorder patients. British Journal of Clinical Psychology. 1990 Nov;29(4):429–32. doi: 10.1111/j.2044-8260.1990.tb00906.x 2289078

[74] Nolen-Hoeksema S, Morrow J. A prospective study of depression and posttraumatic stress symptoms after a natural disaster: The 1989 Loma Prieta earthquake. Journal of Personality and Social Psychology. 1991;61(1):115–21. doi: 10.1037//0022-3514.61.1.115 1890582

[75] Treynor W. Rumination Reconsidered: A Psychometric Analysis. Cognitive Therapy and Research [Internet]. 2003;27(3):247–59.

[76] Nolen-Hoeksema S, Parker LE, Larson J. Ruminative coping with depressed mood following loss. Journal of Personality and Social Psychology. 1994;67(1):92–104. doi: 10.1037//0022-3514.67.1.92 8046585

[77] Isen AM, Daubman KA, Nowicki GP. Positive affect facilitates creative problem-solving. Journal of Personality and Social Psychology. 1987;52(6):1122–31. doi: 10.1037//0022-3514.52.6.1122 3598858

[78] Greene TR, Noice H. Influence of Positive Affect upon Creative Thinking and Problem-solving in Children. Psychological Reports. 1988 Dec;63(3):895–8.

[79] Estrada CA, Isen AM, Young MJ. Positive affect improves creative problem-solving and influences reported source of practice satisfaction in physicians. Motivation and Emotion. 1994 Dec;18(4):285–99.

[80] Fiedler K. Emotional mood, cognitive style, and behavior regulation. In:Fiedler K, Forgas JP. Affect, Cognition, and Social Behavior. Hogrefe & Huber Pub; 1988; 99101–119.

[81] Fiedler K. Toward an account of affect and cognition phenomena using the BIAS computer algorithm In: Forgas JP. Feeling and thinking: the role of affect in social cognition. Cambridge: Cambridge University Press; 2001: pp. 223–252.

[82] Russ SW. Affect and creativity: the role of affect and play in the creative process. Hillsdale, N.J.: L. Erlbaum; 1993.

[83] MacLeod AK, Cropley ML. Depressive future-thinking: The role of valence and specificity. Cognitive Therapy and Research. 1995 Feb;19(1):35–50.

[84] Andersen SM. The inevitability of future suffering: The role of depressive predictive certainty in depression. Social Cognition. 1990;8:203–228.

[85] MacLeod AK, Salaminiou E. Reduced positive future-thinking in depression: Cognitive and affective factors. Cognition & Emotion. 2001 Jan;15(1):99–107.

[86] Andersen SM, Spielman LA, Bargh JA. Future-event schemas and certainty about the future: Automaticity in depressives’ future-event predictions. Journal of Personality and Social Psychology. 1992;63(5):711–23. doi: 10.1037//0022-3514.63.5.711 1447691

[87] Butler G, Mathews A. Cognitive processes in anxiety. Advances in Behaviour Research and Therapy. 1983 Jan;5(1):51–62.

[88] Garber J, Miller SM, Abramson LY. On the distinction between anxiety and depression: Perceived control, certainty, and probability of goal attainment. Human helplessness: Theory and applications. 1980:131–169.

[89] De Raedt R, Koster EHW. Understanding vulnerability for depression from a cognitive neuroscience perspective: A reappraisal of attentional factors and a new conceptual framework. Cognitive, Affective, & Behavioral Neuroscience. 2010 Mar;10(1):50–70. doi: 10.3758/CABN.10.1.50 20233955

[90] Peckham AD, McHugh RK, Otto MW. A meta-analysis of the magnitude of biased attention in depression. Depression and Anxiety. 2013 Mar 6;30(4):407–7. 10.1002/da.20755 21049527

[91] Gotlib IH, Joormann J. Cognition and Depression: Current Status and Future Directions. Annual Review of Clinical Psychology. 2010 Mar;6(1):285–312. doi: 10.1146/annurev.clinpsy.121208.131305 20192795 PMC2845726

[92] Sanchez A, Romero N, De Raedt R. Depression-related difficulties disengaging from negative faces are associated with sustained attention to negative feedback during social evaluation and predict stress recovery. Allen P, editor. PLOS ONE. 2017 Mar 31;12(3):e0175040. doi: 10.1371/journal.pone.0175040 28362826 PMC5376320

[93] Sanchez A, Vazquez C, Marker C, LeMoult J, Joormann J. Attentional disengagement predicts stress recovery in depression: An eye-tracking study. Journal of Abnormal Psychology. 2013 May;122(2):303–13. doi: 10.1037/a0031529 23421524

[94] Noreen S, Ridout N. Short-term memory for emotional faces in dysphoria. Memory. 2010 Jun 7;18(5):486–97. doi: 10.1080/09658211003762092 20544496

[95] Matt GE, Vázquez C, Campbell WKeith. Mood-congruent recall of affectively toned stimuli: A meta-analytic review. Clinical Psychology Review. 1992 Jan;12(2):227–55.

[96] Choi J-W, Lee Y-H.The effects of actual self, ideal self and self-discrepancy on depression. Korean Journal of Clinical Psychology1998;17(1):69–87

[97] Scott L, O’Hara MW. Self-discrepancies in clinically anxious and depressed university students. Journal of Abnormal Psychology. 1993;102(2):282–7. 8315140 10.1037//0021-843x.102.2.282

[98] Strauman TJ. Self-guides, autobiographical memory, and anxiety and dysphoria: Toward a cognitive model of vulnerability to emotional distress. Journal of Abnormal Psychology. 1992;101(1):87–95. doi: 10.1037//0021-843x.101.1.87 1537978

[99] Beck AT. Depression: Clinical, Experimental, and Theoretical Aspects. 1967.

[100] Lemogne C, Mayberg H, Bergouignan L, Volle E, Delaveau P, Lehéricy S, et al. Self-referential processing and the prefrontal cortex over the course of depression: A pilot study. Journal of Affective Disorders. 2010 Jul;124(1–2):196–201. doi: 10.1016/j.jad.2009.11.003 19945172

[101] Auerbach RP, Stanton CH, Proudfit GH, Pizzagalli DA. Self-referential processing in depressed adolescents: A high-density event-related potential study. Journal of Abnormal Psychology. 2015 May;124(2):233–45. doi: 10.1037/abn0000023 25643205 PMC4429006

[102] Beck AT, Rush J, Shaw B, Emery G. Cognitive therapy of depression. New York: Guilford Press; 1979.

[103] Beck AT, Haigh EAP. Advances in Cognitive Theory and Therapy: The Generic Cognitive Model. Annual Review of Clinical Psychology. 2014 Mar 28;10(1):1–24. doi: 10.1146/annurev-clinpsy-032813-153734 24387236

[104] Beck AT. (2002). Cognitive models of depression. In: Leahy RL, Dowd ET, editors. Clinical advances in cognitive psychotherapy: theory and application. New York: Springer Pub. Co; 2002. p29–61.

[105] Marchetti I, Alloy LB, Koster EHW. Breaking the vise of hopelessness: Targeting its components, antecedents and contexts. OSF Preprints. 10.31219/osf.io/ryvwu. 2019. PMC11314313 39131585

[106] Beck AT, Steer RA, Beck JS, Newman CF. Hopelessness, depression, suicidal ideation, and clinical diagnosis of depression. Suicide Life Threat Behav. 1993;23(2):139–145. 8342213

[107] Papageorgiou C, Wells A. Positive beliefs about depressive rumination: Development and preliminary validation of a self-report scale. Behavior Therapy. 2001;32(1):13–26.

[108] Penley JA, Tomaka J, Wiebe JS. The association of coping to physical and psychological health outcomes: a meta-analytic review. J Behav Med. 2002;25(6):551–603. doi: 10.1023/a:1020641400589 12462958

[109] Holahan CJ, Moos RH, Holahan CK, Cronkite RC. Long-term posttreatment functioning among patients with unipolar depression: An integrative model. Journal of Consulting and Clinical Psychology. 2000;68(2):226–32. doi: 10.1037//0022-006x.68.2.226 10780122

[110] Nezu AM, Carnevale GJ. Interpersonal problem-solving and coping reactions of Vietnam veterans with posttraumatic stress disorder. Journal of Abnormal Psychology. 1987;96(2):155–7. doi: 10.1037//0021-843x.96.2.155 3584665

[111] Zacks RT, Hasher L. Directed ignoring: Inhibitory regulation of working memory. In: Dagenbach D, Carr TH, editors. Inhibitory mechanisms in attention, memory, and language. New York NY: Academic Press; 1994. pp. 241–264.

[112] Denckla MB. A theory and model of executive function: A neuropsychological perspective. In: Lyon GR, Krasnegor NA, editors. Attention, memory, and executive function. Baltimore MD: Brookes; 1996. pp. 263–278.

[113] Joormann J, Gotlib IH. Emotion regulation in depression: relation to cognitive inhibition. *Cogn Emot*. 2010;24(2):281–298. doi: 10.1080/02699930903407948 20300538 PMC2839199

[114] Evans J, Williams JMG, O’Loughlin S, Howells K. Autobiographical memory and problem-solving strategies of parasuicide patients. Psychological Medicine. 1992 May;22(2):399–405. doi: 10.1017/s0033291700030348 1615107

[115] Raes F, Hermans D, Williams JMG, Demyttenaere K, Sabbe B, Pieters G, et al. Reduced specificity of autobiographical memory: A mediator between rumination and ineffective social problem-solving in major depression? Journal of Affective Disorders. 2005 Aug;87(2–3):331–5. doi: 10.1016/j.jad.2005.05.004 15979154

[116] Sutherland K, Bryant RA. Autobiographical memory and the self-memory system in posttraumatic stress disorder. Journal of Anxiety Disorders. 2008 Apr;22(3):555–60. doi: 10.1016/j.janxdis.2007.03.008 17467228

[117] Romano M, Moscovitch DA, Ma R, Huppert JD. Social problem-solving in social anxiety disorder. *J Anxiety Disord*. 2019;68:102152. doi: 10.1016/j.janxdis.2019.102152 31704632

[118] Sternheim L, Startup H, Pretorius N, Johnson-Sabine E, Schmidt U, Channon S. An experimental exploration of social problem-solving and its associated processes in anorexia nervosa. *Psychiatry Res*. 2012;200(2–3):524–529. doi: 10.1016/j.psychres.2012.06.029 22809854

[119] Steffen A, Nübel J, Jacobi F, Bätzing J, Holstiege J. Mental and somatic comorbidity of depression: a comprehensive cross-sectional analysis of 202 diagnosis groups using German nationwide ambulatory claims data. *BMC Psychiatry*. 2020;20(1):142. Published 2020 Mar 30. doi: 10.1186/s12888-020-02546-8 32228541 PMC7106695

[120] D’zurilla TJ, Nezu AM. Problem-solving therapy: a positive approach to clinical intervention /. New York, N.Y.: Springer; 2007.

[121] Arean PA, Perri MG, Nezu AM, Schein RL, Christopher F, Joseph TX. Comparative effectiveness of social problem-solving therapy and reminiscence therapy as treatments for depression in older adults. Journal of Consulting and Clinical Psychology. 1993;61(6):1003–10. doi: 10.1037//0022-006x.61.6.1003 8113478

[122] Mynors-Wallis LM, Gath DH, Lloyd-Thomas AR, Tomlinson D. Randomised controlled trial comparing problem-solving treatment with amitriptyline and placebo for major depression in primary care. BMJ [Internet]. 1995 Feb 18 [cited 2019 May 6];310(6977):441–5. Available from: https://www.bmj.com/content/310/6977/441.full doi: 10.1136/bmj.310.6977.441 7873952 PMC2548821

[123] Barrett JE, Williams JW Jr, Oxman TE, Frank E, Katon W, Sullivan M. Treatment of dysthymia and minor depression in primary care: a randomized trial in patients aged 18 to 59 years. J Fam Pract. 2001;50(5):405–412. 11350703

[124] Salkovskis PM, Atha C, Storer D. Cognitive-Behavioural Problem-solving in the Treatment of Patients who Repeatedly Attempt Suicide a Controlled Trial. British Journal of Psychiatry. 1990 Dec;157(6):871–6. 10.1192/bjp.157.6.871 2289097

[125] Gellis ZD, Kenaley B. Problem-Solving Therapy for Depression in Adults: A Systematic Review. Research on Social Work Practice. 2007 Oct 16;18(2):117–31.

