# Supplementary material for: Thinking about the consequences: The detrimental role of future thinking on intrapersonal problem-solving in depression
Source: PLoS One. 2023 Aug 23;18(8):e0289676. doi: 10.1371/journal.pone.0289676 (PMC10446235; doi:10.1371/journal.pone.0289676)
Supplement: S1 Table — (DOCX) [file pone.0289676.s001.docx]

**Supplementary Materials**

Problem scenarios and some examples of resolved and unresolved consequences generated by participants

| **Problem Scenario’s** | **Resolved Consequences** | **Unresolved Consequences** |
| --- | --- | --- |
| **1.** You must prepare an important presentation for university but the more you think about it the more you become worried. | *You become more confident in your ability to given presentations.* | *You become so nervous you end up doing a bad job on the presentation.* |
| **2.** Reflecting upon your life, you feel you are not meeting your expectations. Comparing yourself to others makes you feel inadequate. | *You feel very content with your achievements in life.* | *You end up developing low self-esteem.* |
| **3.** Every time you are stressed you exercise excessively straining yourself. You want to find another way to deal with stress. | *You find another hobby you enjoy which de-stresses you and make some new friends in the process.* | *You end up causing a yourself a permanent injury.* |
| **4.** You have a haircut because you are dissatisfied with your appearance. However, you still don’t like the way you look. | *You feel happier and more confident about your appearance.* | *You say no to going out with friends(socialising) because you don’t feel good about how you look.* |
| **5.** Due to recent tiredness, you decide to eat more healthily. Despite all efforts, you find yourself still eating unhealthy foods. | *You feel good and have a lot more energy to do the things you enjoy.* | *You end up developing diabetes* |
| **6.** You registered to an art class to learn how to paint. You feel insecure and stressed about your first class. | *You sign up for an art trip your class teacher is organising.* | *You end up not attending the class and feeling guilty for wasting your money* |
| **7.** You have been working long hours at work and feel tired. Making things worse, your computer crashes and you lose your work | *Your boss praises you and gives you a promotion* | *You get sacked from your job* |
